# Complete Genome Sequence of *Gordonia* sp. Strain JH63, Isolated from Human Skin

**DOI:** 10.1128/MRA.00059-20

**Published:** 2020-03-12

**Authors:** Kyoung Lee, Sachin Kumar Badaya, Rajni Singh, Jae Yun Lim

**Affiliations:** a Department of Bio Health Science, Changwon National University, Changwon, South Korea; b Amity Institute of Microbial Biotechnology, Amity University, Noida, India; c School of Systems Biomedical Science, Soongsil University, Seoul, South Korea; Loyola University Chicago

## Abstract

We isolated *Gordonia* sp. strain JH63, which has steroid-degrading abilities, from human skin. The complete genome sequence showed that this strain possesses a 5,175,911-bp chromosome and a 162,832-bp plasmid with 4,525 and 159 coding sequences, respectively.

## ANNOUNCEMENT

*Gordonia* is a genus belonging to the family *Gordoniaceae* in the phylum *Actinobacteria*. The strains of this genus have high G+C contents and are Gram positive, rod shaped, and orange pigmented. They are ubiquitously isolated from and detected in metagenomes from the soil, water, sediments, and polluted environments, and some are associated with plants and animals and other living creatures (1, 2). They are reported to degrade various types of recalcitrant and environmental pollutants and to produce bioactive chemicals and a variety of extracellular enzymes (3). Although 44 valid species in the genus *Gordonia* have been reported, many strains remain as unknown species in the NCBI taxonomy database. In this article, we report the isolation of *Gordonia* sp. strain JH63 from human skin and the complete genome sequence of the isolate.

A swab sample from skin of the outer ear of a male college student underwent direct streaking on Difco nutrient agar. The agar medium was incubated for 3 days under aerobic conditions at 28°C. Of the purified isolates, one strain, named JH63, was chosen for its capability to grow in minimal salt basal medium (4) containing 0.5% cholesterol or estradiol as a carbon and energy source at 28°C. *Gordonia* sp. JH63 was identified using 16S rRNA gene sequence analysis and was deposited in the Korean Collection for Type Cultures (KCTC 49188). Ethical approval for subject sampling was granted by the institutional review board of Changwon National University. *Gordonia* sp. JH63 was cultured in nutrient broth at 28°C and 140 rpm shaking for 2 days. Total DNA was extracted using the phenol extraction method (5). The 20-kb genomic library using a total of 5 μg was constructed with a SMRTbell template prep kit 1.0 (catalog number PN 100-259-100; Pacific Biosciences) as previously described (6). The SMRTbell library was sequenced using P6-C4 chemistry (DNA sequencing reagent 4.0) and 1 single-molecule real-time (SMRT) cell using the PacBio RS II sequencing platform (DNA Link, Seoul, South Korea) as previously described (6). The raw reads (977,649,376 bp, 139,007 reads, 7,033-bp mean length) were filtered in SMRT Portal 3.2.0 with 0.75 as the minimum read quality and 50 bp as the minimum read length. The clean data (838,287,123 bp, 116,661 reads), with an *N*_50_ value of 9,340 bp, were used for genome assembly. *De novo* genome assembly was conducted according to the hierarchical genome assembly process workflow with default settings (SMRT Analysis 2.3.0, HGAP2), including consensus polishing using the Quiver consensus algorithm (7). Errors were corrected by mapping short reads (length cutoff, 14,328 bp) onto the longest ~30-fold genome seed bases (150,009,085 bp). Bioinformatics programs such as NUCmer 3.1 and MUMmer 3.5 (8) were used to identify overlapped sequences of both ends for manual genome closure. Gene predictions and annotations were provided by NCBI using the NCBI Prokaryotic Genome Annotation Pipeline 4.10 (9).

The final genome assembly has an average coverage of 96-fold and consists of one circular chromosome of 5,175,911 bp (67.94% G+C content) and one circular plasmid (pJH63-1) of 162,832 bp (65.42% G+C content). The chromosome contains 4,684 coding sequences (CDSs), 3 copies of rRNA genes (5S, 16S, and 23S), and 46 coding regions of tRNAs. The plasmid pJH63-1 contains 159 CDSs. The genes encoding enzymes for the catabolism of cholesterol were identified and categorized into side chain degradation (β-oxidation-like) and steroid nucleus degradation (cholesterol oxidase or 3β-hydroxysteroid dehydrogenase/isomerase, KshA1A2, HsaA1A2, HsaC, and HsaD) (2). The *Gordonia* sp. JH63 genome can contribute to a better understanding of steroid catabolism by skin bacteria.

The BLAST average nucleotide identity (ANIb) values between *Gordonia* sp. JH63 and the most closely related genomes on the databases were calculated using the JSpeciesWS server (10). JH63 shared the highest ANIb with *Gordonia* sp. strain UCD-TK1 (GenBank accession number LZMP00000000) (11), *Gordonia* sp. strain SGD-V-85 (LNQO01000000), and *Gordonia* sp. strain IITR100 (MVKV01000000) (12) at values of 97.31%, 97.12%, and 97.00%, respectively. These values were derived from 89.67, 88.99, and 88.84 aligned percentages, respectively. This result indicated that the ANIb values are above the suggested cutoff (95%) for the delineation of new bacterial species (13), and these strains may form a new species clade of *Gordonia*. Of them, *Gordonia* sp. JH63 represents the first complete genome sequence of this new species in GenBank. The 16S rRNA gene of the JH63 strain showed a 100% sequence similarity to Gordonia terrae NRRL B-16283^T^. However, the ANIb value is 87.23% (76.66% coverage), indicating that *Gordonia* sp. JH63 does not belong to the species *G. terrae*.

### Data availability.

The complete genome sequences of the chromosome and plasmid pJH63-1 of the *Gordonia* sp. JH63 strain were deposited in GenBank under the accession numbers CP047235 and CP047236, respectively. The raw sequencing data have been deposited in the SRA under the accession number PRJNA597702.
